# Molecular epigenetic switches in neurodevelopment in health and disease

**DOI:** 10.3389/fnbeh.2015.00120

**Published:** 2015-05-13

**Authors:** Anke Hoffmann, Christoph A. Zimmermann, Dietmar Spengler

**Affiliations:** Translational Research, Max Planck Society, Max Planck Institute of PsychiatryMunich, Bavaria, Germany

**Keywords:** bivalent domains, NSC, genomic imprinting, transposons, molecular clocks, early-life stress, Avp, polycomb

## Abstract

Epigenetic mechanisms encode information above and beyond DNA sequence and play a critical role in brain development and the long-lived effects of environmental cues on the pre- and postnatal brain. Switch-like, rather than graded changes, illustrate par excellence how epigenetic events perpetuate altered activity states in the absence of the initial cue. They occur from early neural development to maturation and can give rise to distinct diseases upon deregulation. Many neurodevelopmental genes harbor bivalently marked chromatin domains, states of balanced inhibition, which guide dynamic “ON or OFF” decisions once the balance is tilted in response to developmental or environmental cues. Examples discussed in this review include neuronal differentiation of embryonic stem cells (ESC) into progenitors and beyond, activation of *Kiss1* at puberty onset, and early experience-dependent programming of *Avp*, a major stress gene. At the genome-scale, genomic imprinting can be epigenetically switched on or off at select genes in a tightly controlled temporospatial manner and provides a versatile mechanism for dosage regulation of genes with important roles in stem cell quiescence or differentiation. Moreover, retrotransposition in neural progenitors provides an intriguing example of an epigenetic-like switch, which is stimulated by bivalently marked neurodevelopmental genes and possibly results in increased genomic flexibility regarding unprecedented challenge. Overall, we propose that molecular epigenetic switches illuminate the catalyzing function of epigenetic mechanisms in guiding dynamic changes in gene expression underpinning robust transitions in cellular and organismal phenotypes as well as in the mediation between dynamically changing environments and the static genetic blueprint.

## Introduction

The field of molecular epigenetics has evolved rapidly over the past 25 years (Allis, 2009; Armstrong, 2013) and has stimulated more recently a growing interest in the role of epigenetic mechanisms for nervous system function in development, health, and disease (Sweatt, 2013). Major conceptual improvements in our understanding comprise the role of DNA methylation, posttranslational modifications (PTMs) of core histones, nucleosome positioning, and non-coding RNAs (ncRNA) among others (Allis, 2009). Covalent modifications of DNA and chromatin, which packages the DNA into nucleosomes, are at the core of the epigenetic machinery and will be addressed in more detail in this review.

Even so the identification and characterization of these mechanisms has illuminated our insight into the molecular basis of certain epigenetic phenomena, a formal definition of epigenetics remains still a matter of debate. More recently, Adrian Bird suggested that epigenetic events pertain to “the structural adaptation of chromosomal regions so as to register, signal or perpetuate altered activity states” (Bird, 2007). This unifying definition implicitly includes transient as well as stable (i.e., heritable) marks and postulates that epigenetic systems are “responsive, not proactive” by integrating changes imposed by other events.

This broad definition has been criticized by Mark Ptashne (Ptashne, 2013), among others, for describing actually matters of eukaryotic gene regulation. Enzymes that modify DNA and histones are typically recruited by transcription factors (TF) to specific DNA address codes and self-sustaining modifications are postulated to solely apply to DNA methylation, not histones or nucleosomes (Ptashne, 2007, 2014). Epigenetics typically implies a memory process by which a transient signal or event initiates a response that is perpetuated in the absence of the original signal (Ptashne, 2013; Hoffmann and Spengler, 2014). Therefore, histone modifications have been challenged as epigenetic mechanism (Ptashne, 2013), whereas DNA methylation provides an easy-to-understand process for propagating and storing information as cells divide.

A central issue behind this ongoing discussion on the quality of epigenetic mechanisms is causality: are DNA and histone modifications the driving force behind differences in chromatin states and transcription, or are differences in these modifications mostly downstream to dynamic processes such as transcription and nucleosome remodeling (Henikoff and Shilatifard, 2011). As part of the answer to this issue, integrated genome-wide analysis of genetic sequence variation, DNA methylation, chromatin states, transcription rates, and heritability evidenced that sequence variation is a major determinant for the differential recruitment of TF and associated DNA and histone modifications (Furey and Sethupathy, 2013).

Still, many epigenetic phenomena are apparently unrelated to sequence variation. Examples include the peloric variant of toadflax (Cubas et al., 1999), a classic case of what Robin Holliday called epimutation (Holliday, 1991), prion diseases (Tuite and Serio, 2010), the lambda bacteriophage switch between lysis and lysogeny (Ptashne, 2004), and X-chromosome inactivation (Allis, 2009) among others. Such epigenetic changes appear to be less responsive but to fulfill a proactive role in the development of cellular and organismal phenotypes. In due consideration of these findings, Daniel Gottschling proposed that epigenetic phenomena must be switch-like, “ON or OFF, rather than a graded response” and “heritable even if the initial conditions that caused the switch disappear” (Gottschling, 2004).

Prompted by this hypothesis, we will discuss here recent insights into the molecular basis and dynamics of epigenetic switches in the developing and mature brain and briefly refer to their relevance for mental diseases. During the discourse we will consider epigenetic switches at different scales ranging from cells to tissues, from there to physiological systems, and ultimately to their interactions with dynamically changing environments. This said, our discussion is not thought to resolve the “chicken-and-egg situation” related to causality in molecular epigenetics but to illustrate epigenetic mechanisms driving dynamic, switch-like, changes in phenotypes at various neurodevelopmental stages. Lastly, we will reconsider the concept of molecular epigenetic switches in the context of Waddington’s epigenetic landscapes of valleys and mountains where dynamic changes in gene expression drive changes in phenotypes.

## A Shortcut to Epigenetic Marks

The field of epigenetics has been recently treated in a number of comprehensive monographs (Allis, 2009; Tollefsbol, 2011; Armstrong, 2013). Among the various epigenetic mechanisms that are now being recognized, DNA methylation is still the best characterized and is, as a key epigenetic modification, at the focus of this review. Therefore, we will briefly highlight main features of this epigenetic mark and touch on the question how it links to histone marks.

### Methylation Marks in the Book of Life

As far back as to the mid-70th it has been recognized that cytosines in the genome, as part of the genetic code, also undergo chemical modifications of the pyrimidine ring by which a developmental stage- and cell-type-specific epigenetic memory can be directly deposited onto DNA itself (Holliday and Pugh, 1975). DNA methylation refers to the addition of a methyl group to the fifth carbon of cytosine (5mC) (Bird, 2002). In somatic cells, 5mC occurs primarily in the context of palindromic CpG dinucleotides, which are typically methylated in a symmetric manner. Non-CpG methylation (CpH, H = A, T, C) prevails in plants and has been more recently also detected in embryonic stem cells (ESCs) and the mammalian nervous system (Xie et al., 2012) where this modification strongly increases during development from fetal to young adults for reasons still poorly understood (Lister et al., 2013).

The biochemical process of DNA methylation is carried out by a family of enzymes consisting of the DNA methyltransferases DNMT1, DNMT3A, and DNMT3B and the enzymatically inactive homologue DNMT3L (Ooi et al., 2009).

DNMT1, and its obligate partner, the ubiquitin-like plant homeodomain and RING finger domain 1 (UHRF1), which preferentially recognizes hemimethylated DNA (Sharif et al., 2007) act to preserve the methylation profile of the parental DNA strand during replication. In contrast, DNMT3A and DNMT3B catalyze the transfer of methyl groups to unmethylated DNA and act as *de novo* methyltransferases. Hereby their catalytic activity is enhanced by assembly with the homologue DNMT3L as a result of its scaffolding function (Ooi et al., 2009).

The relationship of DNA methylation to gene expression does not match an all-purpose rule, but depends profoundly on the underlying sequence, transcriptional state, and location of the genomic DNA. In this regard, the canonical view of DNA methylation as a repressive mark applies to allele-specific methylation of CpG island (CGI, DNA stretches of high CpG density) promoters of genes on the active copy of the X chromosome vs. the inactive one and imprinting control regions (ICR; Deaton and Bird, 2011).

In contrast, recent genome-wide methylation analysis indicates that most inactive somatic promoters stay unmethylated (Weber et al., 2007) while high amounts of DNA methylation cluster at gene bodies (genomic regions spanning exons and introns) where they associate with increased transcription and/or alternate promoter usage (Weber et al., 2007; Maunakea et al., 2010; Lister et al., 2013). Moreover, gene bodies localizing to partially methylated domains (continuous regions of <70% methylation) show reduced expression when compared to those in highly methylated domains (Lister et al., 2009). The contrast between very low CGI methylation and very high gene body methylation is more pronounced in human brain and neurons (Schroeder et al., 2011) that express high levels of DNMTs (Feng et al., 2005) when compared to non-neuronal cells.

Our previously held view of DNA methylation as a relatively stable, or irreversible, covalent modification that is primarily established during developmental programming has been challenged by the transformative discovery of active demethylation (Wu and Zhang, 2014). Although several oxidation-independent mechanisms have been proposed to contribute to active demethylation (i.e., enzymatic removal of the methyl group from 5mC by MBD2, nucleotide excision repair (NER) to erase 5mC, direct base excision repair (BER) of 5mC by DNA glycosylases, deamination and repair of 5mC base, and radical SAM mechanisms) there is so far no compelling evidence that these biochemical reactions could take place under physiological conditions (Wu and Zhang, 2014). In contrast, the discovery that the ten-eleven translocation (Tet) family of enzymes can catalyze the oxidation of 5mC into 5-hydroxymethylcytosin (5hmC; Tahiliani et al., 2009; Ito et al., 2010), and into further oxidized derivatives [5-formylcytosine (5fC) followed by 5-carboxycytosine (5caC)] (He et al., 2011; Ito et al., 2011), was a breakthrough in unravelling active demethylation processes. The resulting oxidation products (5hmC, 5fC, and 5caC) impair binding and/or activity of the maintenance methylation complex (DNMT1/UHRF1) and lead to replication-dependent passive dilution of oxidized 5mC. This mechanism contributes to demethylation of the paternal genome during preimplantation development and in developing primordial germ cells.

Alternatively, AID/APOBEC proteins may directly deaminate 5hmC to produce 5-hydroxymethyluracil (5hmU)—a pathway suggested to operate in mouse brain (Guo et al., 2011). Subsequently, the DNA glycosylases TDG and SMUG1 are thought to repair the resulting 5hmU:G mismatch (Cortellino et al., 2011).

Oxidized cytosines can be also directly excised by the DNA repair machinery. The derivatives 5fC and 5caC are efficiently eliminated by TDG and repaired by the BER pathway. This mechanism has been described so far only in ESCs (He et al., 2011; Maiti and Drohat, 2011).

It is noteworthy that the oxidized derivatives of 5mC do not behave simply as inert transitional states; 5hmC similar to 5mC is present throughout the genome at specific regulatory regions (Lister et al., 2013) and may counterbalance the functions of 5mC by disrupting and/or restructuring interactions with associated reader and effector proteins (Spruijt et al., 2013).

Together, these findings implicate that, in contradistinction to the concept of DNA methylation being stable, the methylome is dynamic in terms of genomic distribution of 5mC, cyclic enzymatic cascades consisting of cytosine methylation, iterative oxidation, replication dependent dilution, DNA glycosylase-initiated base repair, genomic distribution of oxidized 5mC derivatives, and varying recruitments of readers and effectors.

### Histone Modifications

Chromatin denotes the packaging of genomic DNA, along with histone proteins and associated factors, into a highly condensed form within the nucleus (Misteli, 2007). The nucleosome is the building block of chromatin and consist of 146 bp of DNA wrapped around a histone octamer comprising two copies of each core histone (i.e., H2A, H2B, H3, and H4). Linker histones (i.e., H1) and other non-histone proteins connect nucleosomes and array them into higher order structures largely inaccessible to the transcriptional machinery (Misteli, 2007).

Nucleosomes can physically block the formation of transcription initiation complexes and transcription (Li et al., 2007). On the contrary, various enzymatic complexes can remodel histone-DNA structures to facilitate the recruitment of the transcriptional machinery whereby some of these processes rely on ATP-dependent mobilization of the core histone relative to the DNA to expose regulatory DNA sequences (Cairns, 2007).

A more dynamic function of nucleosomes refers to various PTMs of selected amino acid residues within the free amino-terminal tail of the core histones protruding from the nucleosome surface. These enzymatic reactions include lysine acetylation, lysine and arginine methylation, serine phosphorylation, and covalent binding of ubiquitin among others (Kouzarides, 2007). Enzymatic “writers” of histone marks (e.g., lysine methyltransferases and histone acetyltransferases) are held in check by enzymatic “erasers” (e.g., lysine demethylases and histone deacetylases). The resulting histone modifications give rise to a combinatorial pattern assimilating complex cross-modulatory and hierarchical relationship between single PTMs. A variety of chromatin-binding proteins is capable to read such signatures through specialized domains (e.g., bromodomains and chromodomains) and to recruit additional effector proteins that can re-edit these marks (Kouzarides, 2007). Likewise, a number of DNA-binding proteins recognize and bind to 5mC (e.g., methyl-CpG-binding proteins, Kaiso, Kaiso-like proteins, and SRA domain proteins) and are therefore referred to as “readers” of methylation marks (Fournier et al., 2012). These readers also recruit various effector proteins that regulate DNA methylation and chromatin marks. Accordingly, DNA methylation and chromatin marks can influence each other in deposition and maintenance.

Overall, chromatin structure is very dynamic and subject to alterations at the level of individual histone proteins and nucleosomes, which can serve as a molecular platform for integrating environmental and internal signals at the level of the genome.

Despite this rather packed survey of epigenetic mechanism we feel confident to be appropriately prepared to discuss in the following sections the principal role of molecular epigenetic switches in the developing and mature brain. This said we will commence our discourse with the starting point of any neural cell.

## Molecular Epigenetic Switches in Embryonic Stem Cells

Pluripotent ESCs have the capacity to produce all cell types of an organism. In the course of differentiation the potential for gene expression declines and ultimately results in the commitment to specific cell fates. Exactly how the switch between pluripotent ESCs and their differentiated progeny is regulated has been the subject of intense research (Martello and Smith, 2014).

**Figure 1:**
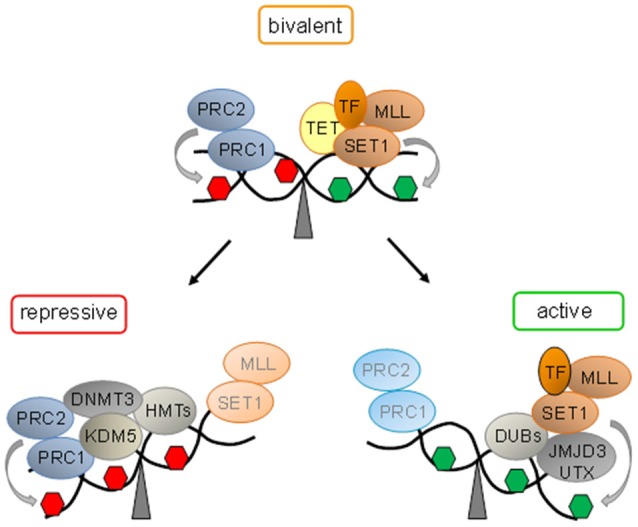
**Bivalent domains underlie epigenetic switches in stem cells**. At bivalent domains the simultaneous presence of repressive (red hexagon) and active (green hexagon) chromatin marks counterbalance each other. Polycomb complexes (PcG, comprising PRC1 and PRC2) confer repression by H3K27me3 catalyzed by the subunit Suz12. Ten − eleven translocation proteins (Tet) interact with bivalent domains and catalyze DNA demethylation. Activating transcription factors (TF) together with histone H3K27 demethylases (SET1 and MLL) and H2. A-deubiqitinating enzymes (DUBs) tilt the balance towards activation and replacement of repressive protein complexes. Conversely, loss of activating protein complexes switches bivalent domains into a repressed state by recruitment of H3K4 demethylase (KDM5). Histone methyltransferases (HMT) mediated H3K9 methylation and *de novo* DNA methyltransferase (DNMT3) mediated DNA methylation lock in repression.

The Trithorax group (trxG) and Polycomb group (PcG) proteins play important roles in early development due to their chromatin-modifying activities (Schuettengruber et al., 2007). A subset of trxG proteins—comprising SET1A, SET1B, and mix lineage leukemia (MLL) proteins 1–4 in mammals (Shilatifard, 2012) catalyze trimethylation of histone H3Lys-4 (H3K4me3), an activating mark. In contrast, PcG proteins confer silencing by formation of the Polycomb-repressive complexes (PRCs) 1 and 2, whereby the enzymatic subunit of PRC2, the histone methyltransferase Suz12, catalyzes H3K27me3, a hallmark of repressive chromatin (Simon and Kingston, 2013; Figure 1).

The genome-wide distribution of H3K4me3 and H3K27me3 was firstly analyzed by chromatin immunoprecipitation (ChIP) and DNA tiling arrays in mouse ESCs and resulted in the enrichment of various groups of developmental genes including the *Hox* cluster (Bernstein et al., 2006). Most transcriptional start sites (TSSs) associated with H3K4me3, whereas the distribution of H3K27me3 was broader and unexpectedly spanned ~75% of the TSSs that were also marked by H3K4me3. Interestingly, sequential ChIP experiments indicated the coexistence of these opposing histone marks at select genes, which typically encoded developmental TF. These so called bivalent genes showed low expression in the undifferentiated state. Following neuronal differentiation, some bivalent genes underwent expression concomitant with the departure of H3K27me3, while others undergoing silencing lost H3K4me3 but retained H3K27me3. These findings inspired the intriguing hypothesis that bivalent domains serve to keep developmental genes on standby primed for subsequent expression and to defend against unscheduled expression (Figure 1). Collectively, bivalent domains are thought to reduce transcriptional noise in favor of robust developmental decisions (Bernstein et al., 2006).

Genome-wide analysis by combining ChIP with next-generation sequencing (ChIP-seq) showed that almost all promoter CGIs of high CpG density were marked by H3K4me3, whereby ~22% contained at the same time H3K27me3, and were weakly expressed (Mikkelsen et al., 2007). Following differentiation into neuronal progenitors, bivalent states declined by ~92% indicating that some bivalent domains may persist and/or reoccur in nonpluripotent cells.

In support of this hypothesis, differentiation of mouse ESCs into radial glial neuronal precursor cells (RGCs) caused the resolution of ~675 bivalent domains concomitant with the formation of ~550 new ones (Mohn et al., 2008). Upon terminal differentiation, ~1000 bivalent domains were resolved, while ~340 ones were restored. Together, these results suggest that bivalent domains are not restricted to a specific stage but evolve dynamically across development.

One clue to the formation of bivalent domains arose from the analyses of their underlying DNA sequences. Bivalent domains strongly correlate with CGIs (Bernstein et al., 2006) and virtually all CpG-rich promoters in ESCs are methylation free (Weber et al., 2007). The methyltransferases MLL1 and MLL2 as well as the zinc finger protein Cfp1 (a subunit of SET1A/B) initiate the formation of bivalent domains upon DNA-binding via the so-called zinc finger CXXC domain that specifically recognizes unmethylated CpG islands. Similarly, TET1 and TET3 proteins localize to bivalent domains via their CXXC domains while TET2 is recruited indirectly by the CXXC domain of IDAX (Ko et al., 2013). Together, TET proteins act to maintain DNA hypomethylation at bivalent domains. The precise order of events leading to the establishment and less-known resolution of bivalent domains is beyond the scope of this review and still subject to intense research as reviewed elsewhere (Voigt et al., 2013).

Overall, bivalent domains provide persuasive evidence for molecular epigenetic switches in gene expression in ESCs and beyond. The simultaneous presence of active and repressive marks and associated complexes keeps bivalent loci in a state both responsive to developmental cues and at the same time refractory to subthreshold noise. Once this dynamic equilibrium between activation and repression is tilted towards differentiation, switch-like, robust “ON or OFF” decisions, rather than graded responses, take place. As a result bivalent domains give way to gene silencing or expression in a cell-lineage and/or cell-type specific fashion (Figure 1).

## Molecular Epigenetic Switches in Genomic Imprinting

According to Mendel’s 2nd law, separate genes for separate traits are inherited independently of each other from parents to offspring and result in equivalent complements of paternally and maternally expressed autosomes in mammals (Speicher et al., 2010). Still, distinct autosomal regions on the paternal or maternal allele can be selectively silenced in a heritable manner dependent on the parental origin. This process, termed genomic imprinting, is initiated in the corresponding parental germ cells, escapes from genome-wide demethylation during fertilization and preimplantation, and is thought to be stably maintained across an organism’s lifespan. About 150 genes with a verified imprinting status have been identified in mice (Jirtle, 2012) with many of them being conserved in human (Morison et al., 2005).

A variety of molecular mechanisms including DNA methylation, ncRNA, and chromatin modifications, among others, are thought to interact in establishing monoallelic expression (Edwards and Ferguson-Smith, 2007). Parental-origin specific DNA methylation of confined, so-called differentially methylated regions (DMR), is instrumental in priming and maintaining genomic imprinting.

In expansion of the genetic conflict theory of genomic imprinting (Moore and Haig, 1991), imprinted genes have been postulated to mediate the “battle-of sexes” in fetal growth control between constrictive maternal and promotive paternal genes, respectively (Constância et al., 2004). The “imprinted brain” theory (Badcock and Crespi, 2008) holds that this imbalance may also apply to the development of brain architecture, cognitive and neuroendocrine functions, and associated neurodevelopmental and/or psychiatric diseases, whereas the related coadaptation theory suggests that paternally expressed genes might be subject to silencing because maternally expressed genes operate complementarily in the pup and the mother (Peters, 2014).

In spite of these varying models, the cellular and molecular mechanisms through which imprinted genes influence neuronal functions remain so far poorly understood. Recent studies on the roles of imprinted genes in NSCs provide, however, new clues to their function and an unexpected flexibility in genomic imprinting (Hoffmann et al., 2014; Daniel et al., 2015).

The protein-coding genes delta-like homolog (*Dlk1*) and type III iodothyronine deiodinase (*Dio3*) are both expressed from the paternal inherited allele and map on chromosome 14q32 and distal chromosome 12 in human and mice, respectively (da Rocha et al., 2008). In contrast, several imprinted large and small ncRNA genes are expressed from the maternal inherited allele. Genetic defects at the *Dlk1-Dio3* locus cause developmental and growth anomalies in various tissues comprising placenta, cartilage, skeletal muscle, and bone (da Rocha et al., 2008).

**Figure 2:**
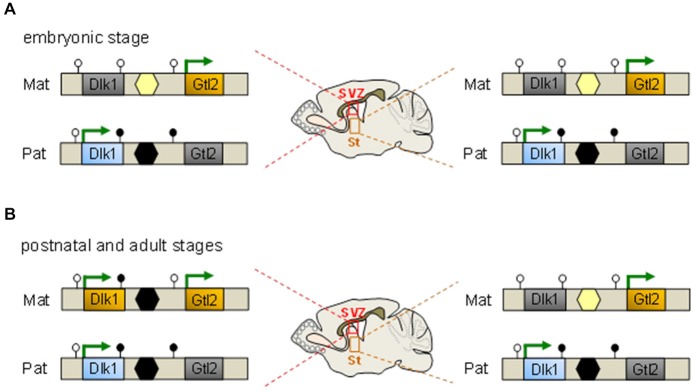
**An epigenetic switch controls *Dlk1* imprinting. (A)** Throughout embryonic development *Dlk1* is monoallelically expressed from the paternal allele in multiple neuronal tissues comprising the subventricular zone (SVZ) and striatum (St) among others and in non-neuronal tissues. Maternal silencing correlates with the absence of germline-derived DNA methylation at the intergenic differentially methylated regions (DMR) (yellow hexagon) which resides between the *Dlk1* and *Gtl2* genes and the absence of somatic methylation of the DMRs at the same genes (unfilled lollipops). Conversely, paternal expression associates with germline-derived methylation at the intergenic DMR (black hexagon) in concert with somatic methylation at the downstream DMRs at *Dlk1* and *Gtl2* (filled lollipops). Methylation patterns are identical in the SVZ (left scheme) and St (right scheme) at embryonic age. **(B)** *Dlk1* is biallelically expressed in the SVZ but not in the St from postnatal day 7 onward and in adulthood. The paternal methylation pattern (i.e., methylation at the downstream *Dlk1* DMR, the intergenic DMR but not the *Gtl2* DMR) is largely adopted by the expressed maternal allele. At opposite, monoallelic Dlk1 expression together with the underlying methylation pattern are maintained in the St.

The reciprocally imprinted *Dlk1* and *Gtl2* (gene trap locus 2, also known as maternally expressed gene 3, *Meg3*) genes map 80 kb apart and flank an intergenic DMR consisting of tandem repeats that represent the only region at the domain marked by germ line-derived paternal methylation (Figure 2). Two additionally DMRs (downstream to *Dlk1* and upstream to *Gtl2*) show hypermethylation on the paternal and hypomethylation on the maternal allele, respectively (Takada et al., 2002).

The broad and high expression of Dlk1 during development declines with maturation but still persists in the subventricular zone (SVZ), particularly in NSCs and astrocytes of the germinal niche, but not in their differentiated derivatives such as neuroblasts and parenchymal astrocytes. Astrocytes residing in the SVZ predominantly produce the secreted form of Dlk1, whereas NSCs predominantly contain the membrane bound form of Dlk1. Co-culture with naïve, but not with Dlk1-deficient astrocytes enhances NSC proliferation indicating that both forms are important for neurogenesis (Ferrón et al., 2011).

Mice lacking *Dlk1* show an increased proliferation of NSCs at early postnatal stages, a subsequent reduction in the number of quiescent NSCs in adults, and depletion at old age. Unexpectedly, this outcome is unrelated to the parental origin of the mutated *Dlk1* allele predicting that both copies contribute to neurogenesis (Ferrón et al., 2011). In fact, both copies of Dlk1 are expressed from postnatal day 7 onward in NSCs and niche astrocytes (Figure 2) concomitant with increased DNA methylation at the germline regulated DMR on the maternal allele. In contradistinction, genomic imprinting is detected at all embryonic stages and is preserved in adult brain cells unrelated to SVZ (Ferrón et al., 2011).

Taken together, these findings exemplify how genomic imprinting can be switched off during confined developmental time windows in specific neuronal cell types and indicate an unprecedented flexibility of this epigenetic mechanism. Transient genomic imprinting in NSCs is not restricted to *Dlk1-Dio3*, but has been also suggested to *Igf2-H19* and *Rasgrf1* in very small embryonic-like stem cells (Hoffmann et al., 2014; Daniel et al., 2015). Collectively, molecular epigenetic ON and OFF switches at imprinted genes may provide a versatile mechanism for dosage regulation of genes with an important role in balancing stem cell quiescence vs. proliferation.

## Molecular Epigenetic-like Switches in Neuronal Progenitor Cells

Transposable elements were firstly discovered in the 1940s by Barbara McClintock in maize and designated as “controlling elements” that in response to certain stressors migrate along chromosomes or “transpose” in the genome and subsequently turn genes on and off in their new location (McClintock, 1951). Sequence analysis evidenced the repeat nature of these elements that unexpectedly make up almost half of mammalian genomes (for comparison; protein coding genes account for less than 2%)(Lander et al., 2001). Once misjudged as “junk” or “selfish” DNA (Dawkins, 2006), new interest has been raised by the ENCODE and FANTOM genome projects, which showed that transposable elements are highly active in regulating their own cell-specific transcription together with the one of adjacent genes (Faulkner et al., 2009; Djebali et al., 2012; Thurman et al., 2012).

DNA transposons invade via a “cut-and-paste” mechanism into new territories though they have disappeared from the genomes of higher eukaryotes, whereas RNA transposons (so-called retrotransposons) migrate via a “cut-and-copy” mechanism that leads to the replication and insertion into new places in the genome (Levin and Moran, 2011). Retrotransposons can be distinguished into long-terminal repeat (LTR) or non-LTR elements; the latter are still active in human genomes and comprise long and short interspersed nuclear elements (LINEs and SINES, respectively).

Transposable elements meet essential criteria of an epigenetic mechanism (responsive to the environment, self-sustaining in the absence of the initial stimulus, and transcriptionally active) although they disrupt genomic nucleotide sequence (Bird, 2007) a limitation acknowledged by the designation “epigenetic-like”.

The existence of mobile elements is not new although they were thought to be active only in germ cells, pluripotent cells, and cancer tissues. Therefore, the recent discovery that they increase their activity during the differentiation of neural progenitor cells (NPCs) into neurons came as a surprise (Muotri et al., 2005). The advent of new techniques (retrotransposition reporter assays and next-generation sequencing) evidenced that somatic retrotransposition is higher in neurons and human tissue culture models of neural development than compared to other somatic tissues (Muotri et al., 2005; Coufal et al., 2009; Bundo et al., 2014), enhanced in Rett syndrome and translational mouse models (Muotri et al., 2010), and ataxia telangiectasia (Coufal et al., 2011). Estimates based on qPCR copy number assays indicated 80–800 new insertions in each hippocampal neuron (Coufal et al., 2009), whereas newer single-cell sequencing data suggest a rate of unique somatic insertions <0.6 insertions per neuron in the caudate or cortex (Evrony et al., 2012). Still, this number corresponds to 1 insertion per 300 cells amounting to more than 100 million unique somatic insertions into the human brain (Reilly et al., 2013).

**Figure 3:**
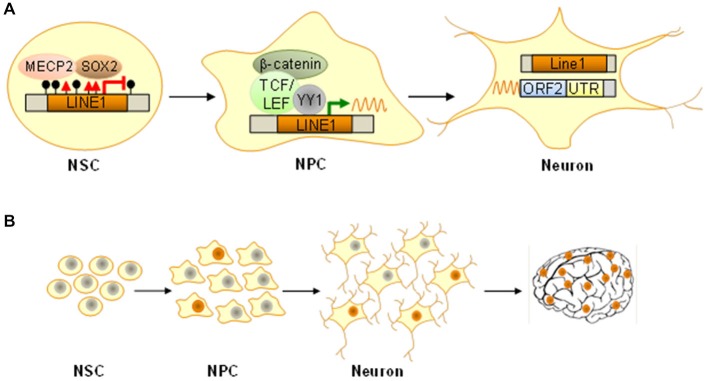
**Epigenetic-like switches in NPCs. (A)** In NSCs the long interspersed nuclear element 1 (LINE1) is silenced by histone H3K9 trimethylation (red triangles), DNA methylation (filled lollipops), and binding of the methyl-CpG-binding protein 2 (MECP2) in concert with the transcription factor SOX2. Following differentiation into NPCs, SOX2 and MECP2 dissociate and thus facilitate formation of open chromatin together with LINE1 demethylation. Wingless-dependent β-catenin-mediated activation of TCF/LEF TF, possibly in cooperation with the transcriptional regulator YY1, induces LINE1 transcription and active retrotransposition, which persists in mature neurons (ORF2, open reading frame 2; UTR, untranslated region). **(B)** Somatic *de novo* insertions in NPCs are maintained as they differentiate into mature neurons with unique genomes. Dependent on developmental stage and temporospatial trajectories such genomic mosaicism can expand from few cells to sizeable populations underlying distinct circuitries and/or structures and ultimately manifest with altered function.

Regulation of LINE1 expression is necessary for transcribing it into an RNA intermediate and subsequent mobilization. The canonical LINE1 promoter harbors binding sites for SOX2 (Tchénio et al., 2000), YY1 (Athanikar et al., 2004), RUNX3 (Yang et al., 2003), TCF and LEF (Kuwabara et al., 2009) all of these TF are expressed from bivalent marked promoters in NPCs and play a pivotal role in neurogenesis. In the undifferentiated state, repressive chromatin, SOX2 (SRY-related HMG-box gene 2), and MECP2 (methyl-CpG-binding domain protein 2) work together in LINE1 repression (Muotri et al., 2005, 2010; Figure 3A). Differentiation elicits SOX2 downregulation, DNA demethylation, MECP2 dissociation and consequently LINE1 derepression (Figure 3A). Concomitantly, the onset of wingless signaling drives TCF/LEF dependent transactivation of LINE1 expression as NPCs differentiate into neurons (Figure 3A).

The question remains why evolution has not erased these remnants of ancient viruses from our genomes given that mobile elements have a high chance of introducing potentially harmful genetic flaws. Possibly, somatic retrotransposition serves to increase stochastically neuronal diversity expanding thus the variance of cellular and organismal phenotypes particularly well suited to the tasks the brain will confront (Figure 3B). A static somatic genome is unable to change or respond to “unanticipated challenges that are not precisely programmed” though “they are sensed and the genome responds in a discernible but initially unforeseen manner” (McClintock, 1984).

Although controlled retrotransposition is hypothesized to benefit neuronal genomes, recent evidence from patients and animal models caution that transposon misregulation may contribute to various mental disorders including RTT (Muotri et al., 2010), schizophrenia (Bundo et al., 2014) and stress-related diseases like posttraumatic stress disorders (PTSD; Ponomarev et al., 2010) and major depression (Hunter et al., 2012).

Overall, retrotransposition represents a molecular epigenetic-like switch in neural progenitors occurring stochastically and/or in response to genomic stress to increase neuronal diversity, and possibly, flexibility regarding novel demands. Hereby, retrotransposition activation acts in tandem with rapid epigenetic switches at bivalent promoters of genes with a role in NPC maintenance vs. differentiation.

## A Molecular Epigenetic Switch in Puberty

Puberty is a critical transition phase in life characterized by the transformation of physical traits that signal the acquisition of reproductive capacity and an extensive structural and functional reorganization of the brain (Crone and Dahl, 2012).

The most important event in the onset of puberty is the release of substantial amounts of gonadotropin-releasing hormone (GnRH) in a pulsatile mode during sleep, which triggers re-awakening of the hypothalamic-pituitary-gonadal (HPG) axis. Contrary to widely held believe, this reproductive axis is first active during prenatal and early postnatal life, a period known as neonatal “mini-puberty”, to be turned off and stay dormant throughout childhood via inhibitory inputs to the hypothalamus (Melmed and Williams, 2011). Hence, puberty does not implicate the developmental maturation of the HPG axis since this system is already primed and under tonic inhibition long before puberty.

GnRH expressing neurons localize predominantly in the preoptic area and the hypothalamus and sent their axons to the median eminence, where they release GnRH in a pulsatile manner into the hypophyseal-portal system. At the anterior pituitary, GnRH stimulates release of luteinizing hormone (LH), which is transported by the bloodstream to the gonads and stimulates steroidogenesis (Melmed and Williams, 2011).

Even though GnRH neurons are under tonic inhibition by GABAergic innervation and locally produced enkephalins, release from this brake does not suffice to elicit puberty indicating the need for further activational signals (Melmed and Williams, 2011). In this respect, the discovery of the small peptide hormone kisspeptin has strongly advanced our insight into the control of reproduction, energy expenditure, and food intake. This small RF-amide neuropeptide (peptides characterized by a common carboxyl-terminal arginine (R) and an amidated phenylalanine (F) motif) is encoded by the *Kiss1* gene and binds with high affinity to the receptor KISS1R. Neurons expressing kisspeptin reside in the arcuate nucleus and the anteroventral periventricular nucleus, two brain regions with an important role in the control of GnRH activity, including GnRH secretion, LH release, and puberty (Piet et al., 2015). Still, the question remains which molecular mechanisms switch on neuronal kisspeptin expression?

Recent evidence suggests that epigenetic mechanisms play a crucial role in guiding pubertal brain development (Lomniczi et al., 2013). Pharmacological treatment of prepubertal female rats with 5-azacytidine (5-aza), an inhibitor of DNA- and RNA-methyltransferases, postponed significantly puberty onset. Animals treated by 5-aza weighted more than controls disfavoring indirect effects related to a loss of body weight. On the other hand, ovaries appeared underdeveloped but without obvious histopathological abnormalities and responded well to gonadotropin treatment. Similarly, the pituitary and medial basal hypothalamus of 5-aza treated rats were efficiently stimulated by GnRH and kisspeptin, respectively, pointing to a central deficit in the availability of kisspeptin (Lomniczi et al., 2013).

Methylation array analysis of hypothalamic DNA before, during, and just after puberty resulted in an enrichment of genes regulating chromatin and histone modifications with several of these belonging either to the PcG silencing complex (*Cbx7*, *Cbx8*, *Eed, Phc3*, *Yy1*, and *Rnf2*) or interacting partners (*Rybp*, *Csnk2b*, *Kdm2b*). All of these genes, except *Rnf2*, were subject to increased methylation at puberty indicating that the PcG complex undergoes functionally relevant epigenetic changes with the onset of puberty and that this repressive gene complex is a key factor in mediating central inhibition. At the same time, methylation at the *Kiss1* promoter stayed unaltered (Lomniczi et al., 2013).

**Figure 4:**
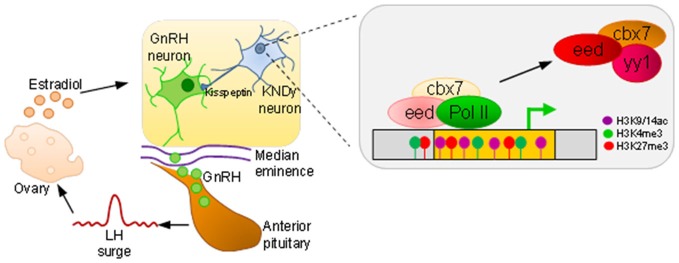
**An epigenetic switch in puberty**. At puberty onset, GnRH expressing neurons, residing in the preoptic area and hypothalamus, release GnRH in a tightly controlled fashion from their axon terminals into the median eminence. The capillary plexus in the upper infundibulum transports GnRH to the anterior pituitary to stimulate luteinizing hormone (LH) secretion. A surge in LH release is necessary to induce ovulation, and subsequent estradiol production feeds back to GnRH neurons. Kisspeptin derived from kisspeptin/neurokinin B/dynorphin (KNDy) expressing neurons stimulate GnRH neurons. During prepubertal age, polycomb complexes (comprising the factors eed, cbx7, and yy1) occupy *Kiss1* and confer silencing. As puberty draws near, dynamic hypermethylation of *eed* and *cbx7* leads to a decrease in repressive (H3K27me3) histone marks in favor of an increase in active ones (H3K9/14ac, H3K4me3) and tilts balanced inhibition towards *Kiss1* activation, polymerase II (PolII) driven transcription, and puberty onset.

Interestingly, analysis of histone modifications at the *Kiss1* promoter evidenced the presence of the repressive mark H3K27me3 (a hallmark of PcG complexes) concomitantly with the active marks H3K4me3 and H3K9, 14ac thus matching the criteria of a bivalent promoter (Figure 4). Moreover, both activation marks increased at the initiation of puberty during the late juvenile phase whereas the repressive mark did not decline until puberty was established (Lomniczi et al., 2013).

Consistent with a role of PcG complexes in *Kiss1* regulation, the 5-aza responsive genes *Eed* and *Cbx7* were expressed in kisspeptin positive neurons. Hereby, Eed occupied the *Kiss1* promoter, suppressed gene expression (Figure 4), and delayed puberty onset following site-directed expression in the hypothalamus (Lomniczi et al., 2013). In accord with this scenario, 5-aza treatment prevented timely *Eed* eviction and the establishment of activating marks at the *Kiss1* gene.

Taken together, these findings suggest a tightly regulated system of balanced inhibition in the control of puberty onset. PcG binding and bivalent marking of *Kiss1* keeps this central driver primed for activation. Puberty induced hypermethylation of *PcG* genes results in their down regulation, loss of PcG-mediated repression, and tilts the balance towards *Kiss1* activation. In conclusion, the neuroendocrine control of female puberty involves the participation of repressive PcG complexes acting as a brake on the HPG axis that must be released for puberty to proceed (Figure 4).

## Plastic DNA Methylation (re-) Sets Seasonal and Circadian Clocks

Seasonal timing of reproduction is common in many temperate-zone vertebrates and refers to the precise coordination of behavioral, neural, and hormonal systems in response to steady changes in day length. The light/dark cycle entrains an endogenous circadian rhythm in nocturnal pineal melatonin (MEL) secretion, that in turn, controls the reproductive neuroendocrine system (Reiter, 1980). Molecular mechanisms mediating the switch between longer and shorter duration MEL signals—triggering gonadal involution and reactivation, respectively—are still incompletely understood. Hypothalamic thyroid hormone (T_4_) signaling has been suggested to play a critical role to translate photoperiodic information into the reproductive neuroendocrine system even so thyroid secretion of the prohormone T_4_ is unaltered by seasonal change (O’Jile and Bartness, 1992). In contrast, hypothalamic expression of deiodinase enzymes that catalyze the conversion of T_4_ into the receptor-active triiodothyronine (T_3_) via deiodinase type 2 (DIO2) or the receptor-inactive enantiomer via deiodinase type 3 (DIO3) are tightly regulated by changes in photoperiod (Barrett et al., 2007; Ono et al., 2008). In this regard, shorter day length triggers increased *dio3* expression, curtails T_3_ signaling, and inhibits gonadotropin secretion. Contrarily, longer day length triggers increased *dio2* expression, promotes T_3_ signaling, and stimulates gonadotropin secretion.

Prompted by these findings, a recent study investigated whether photoperiod- and MEL-driven switches in hypothalamic *dio3* expression couple to epigenetic mechanisms (Stevenson and Prendergast, 2013). In accord with this hypothesis, exposure to inhibitory winter periods in long-day (summer) breeding hamster reduced hypothalamic Dnmt expression and proximal *dio3* promoter methylation concomitantly with enhanced *dio3* expression. Furthermore, pharmacological blockade of photoperiod-driven *dio3* demethylation mitigated reproductive responses to winter photoperiods. Conversely, spontaneous anticipation of spring initiated *dio3* remethylation, decreased dio3 mRNA expression, and paved resumption of reproductive behavior.

Collectively, this work shows that photoperiod- and MEL-dependent changes in Dnmt expression couple to iterative cycles of demethylation and remethylation of the *dio3* promoter. This epigenetic switch concurs with corresponding changes in *dio3* expression controlling T_3_ signaling, and ultimately, gonadal regression and recrudescence.

The circadian clock machinery consists of a far-reaching network of timing mechanisms that serve to maintain the homeostatic balance of an organism. The central master clock sets the pace for peripheral clocks via a number of output signals that synchronize the system as a whole and consists of ~20,000 pacemaker neurons in the suprachiasmatic nucleus (SCN; Takahashi et al., 2008). These pacemakers are entrained by light—a critical *zeitgeber* (time-giver)—via the retinohypothalamic tract and direct circadian rhythms in a number of peripheral tissues. Various nutrient sensors further calibrate peripheral clocks by coupling metabolic states to histone modifications regulating clock activity (Katada et al., 2012).

**Figure 5:**
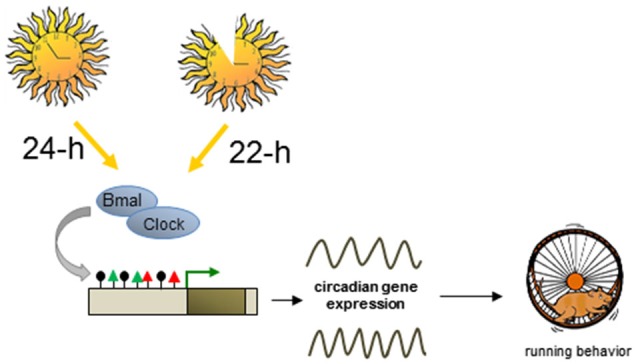
**Plastic DNA methylation mediates switches in circadian cycles**. Altered lightning environment (22-h instead of 24-h days) induces enduring changes in the genetically determined period of circadian behavior in adult mice. Circadian and non-circadian genes downstream to the core TF Bmal and Clock undergo dynamic changes in DNA methylation (black lollipops) and histone modifications (red and green triangles) leading to altered transcriptional output and changes in mouse behavior.

Circadian clockworks incorporate intricate transcription-translational feedback cycles of a group of evolutionary conserved “clock” genes (Takahashi et al., 2008). The activating transcriptional input consists of the core TF CLOCK and BMAL, which stimulate circadian gene expression. Major targets include period (*PER*) and cryptochome (*CRY*) family members representing the negative regulatory output of the circadian clock system. Chromatin remodelers and histone modifications act in concert with transcriptional cycles in establishing the oscillation pattern of molecular clocks (Gallego and Virshup, 2007). Here we will address the question whether epigenetic events can also serve to (re-) set the timing of molecular circadian clocks (Figure 5).

The timing of daily circadian behavior is highly variable among different individuals, and twin studies have indicated that about half of this variability is environmentally controlled. While seasonal light input is known to acutely reorganize the mature biological clock, recent evidence suggests that it can also lastingly program the developing mammalian clock, associated behavior, and switch the response to subsequent seasonal change under seasonal light cycles (Ciarleglio et al., 2011).

Interestingly, a further study showed that altered lighting environment (22-h instead of 24-h days) can also enduringly change the genetically determined period of circadian behavior in adult mice (Azzi et al., 2014). Transcriptome sequencing from the SCN of mice entrained for 4 weeks to 22-h or 24-h days confirmed changes in clock gene expression, notably *Per* genes and *Cry2*, but also globally across the transcriptome at both circadian and non-circadian genes encoding various aspects of neuronal function, chromatin, and DNA modification such as Dnmts and the Tet family. Prompted by these results the authors investigated whether modifications in DNA methylation may underlie changes in circadian rhythms. Mapping of immunoprecipitated methylated DNA (MeDIP) to genome-wide promoter tiling arrays evidenced significant changes between mice entrained to 22-h vs. 24-h days. These changes included a total of 1,294 differentially methylated (hypo- or hyper-methylated) promoter regions of genes relevant to synaptogenesis, axonal guidance, and neurohormone signaling, which correlated with changes in clock genes and global gene transcription (Azzi et al., 2014).

It is noteworthy that infusion of a DNA methyltransferase inhibitor near the SCN during the entrainment to a 22-h light-dark cycle decreased global SCN methylation levels and inhibited the shift in behavioral activity compared with vehicle-treated 22-h mice. Moreover, despite being stable for weeks after termination of the entrainment, light-induced methylation changes were also reversible when 22-h mice were re-entrained to a 24-h light-dark cycle.

In conclusion, light entrainment can reset genetically preset circadian cycles and is reversible by anew entrainment (Figure 5). Entrainment to shorter light-dark cycles induces plastic changes in DNA methylation, associated gene expression in the SCN, and behavioral adjustment to the light-dark cycle and thus demonstrates an unexpected flexibility of DNA methylation in switching between these different states.

## Molecular Epigenetic Switches in Early-Life Stress Dependent Programming of *Avp*

A body of studies over the last years has provided compelling evidence that various environmental conditions (Bird, 2002), including social experiences (Murgatroyd et al., 2010; Szyf and Bick, 2013), can induce enduring epigenetic effects on phenotype (Hoffmann and Spengler, 2014; Zannas and Binder, 2014). Here, we will discuss recent findings showing that epigenetic switches at the *Avp* gene—encoding the neuropeptide arginine vasopressin (Avp)—play an important role in epigenetic programming of the hypothalamic-pituitary-adrenal (HPA) axis, the major stress system in mammals.

Early-life adversity denotes different situations such as parental maladjustment (violence, criminality, substance abuse, and mental illness), interpersonal loss (parental death or divorce and other separation from parents/caregivers), and maltreatment (physical or sexual abuse and neglect) among others (Green et al., 2010).

Epidemiological data support a strong correlation between childhood adversities and mental disease in adulthood (Felitti et al., 1998; Edwards et al., 2003) and epigenetic mechanisms seem to mediate, at least in part, the effects of adverse exposures on future wellbeing (Hoffmann and Spengler, 2012; Raabe and Spengler, 2013; Patchev et al., 2014).

Early-life adversity frequently elicits HPA-axis activation and results in enhanced glucocorticoid secretion, which has to be set back to resting conditions to prevent the deleterious effects of sustained levels on depression and anxiety (Heim et al., 2008). Different brain regions perceiving various stressors such as ELS signal to the paraventricular hypothalamus (PVN) to release AVP and corticotropin-releasing hormone (CRH). These neuropeptides stimulate the expression of pituitary proopiomelanocortin (POMC) and the secretion of its post-translational product adrenocorticotropin (ACTH). Subsequently, ACTH triggers the production of glucocorticoids from the adrenal glands, which upon binding to nuclear glucocorticoid and mineralocorticoid receptors in the limbic system and anterior pituitary; reset HPA-axis activity in response to stress.

To investigate the molecular events that initiate ELS-sensitive *Avp* methylation (Murgatroyd et al., 2009) (see below), we differentiated ESCs into neuronal progenitors and hereafter into dorsal-like hypothalamic cells coexpressing the neuropeptides Avp and oxytocin (Murgatroyd and Spengler, 2014).

The downstream *Avp* enhancer region plays an important role in cell-type specific expression and ELS-dependent epigenetic programming (Murgatroyd et al., 2009). This region was largely methylation free in undifferentiated Avp-negative ESCs, but underwent robust methylation in hypothalamic-like Avp-expressing neurons. A similar pattern was also detected in embryonic and postnatal mice PVNs. Together; these findings agree with the hypothesis that DNA methylation is not an all-purpose mechanism of gene repression and can associate with gene activation outside core promoter regions.

**Figure 6:**
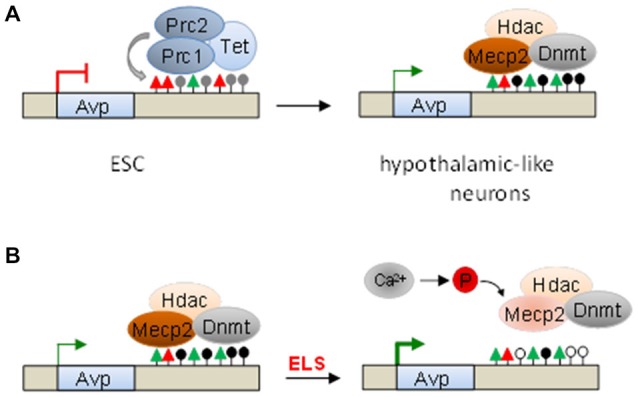
**Serial epigenetic switches underlie early-life stress (ELS)-dependent programming of *Avp*. (A)** Polycomb complexes (Prc1 and Prc2) occupy the downstream *Avp* enhancer in undifferentiated embryonic stem cells (ESC). The histone methyltransferase Suz12, the catalytic subunit of Prc2, and ten-eleven translocation (Tet) proteins catalyze H3K27me3 (red triangles) and 5hmC (gray lollipops), respectively. Upon hypothalamic-like differentiation, PcG and Tet proteins dissociate, while *de novo* DNA methyltransferases (Dnmt) enter and promote *Avp* enhancer methylation. This allows binding of the methyl-CpG-binding protein 2 (Mecp2) and associated repressor complexes, which modulate Avp expression. **(B)** ELS-driven neuronal activity triggers calcium-calmodulin kinase-dependent Mecp2-S421 phosphorylation, enhancer dissociation, and *Avp* derepression. Loss of Mecp2 and associated Dnmts tilts the balance between methylation and postnatal methylome reconfiguration towards demethylation thus leaving an enduring memory trace of the initial event.

To assess which alternative mechanisms silence *Avp* in non-neuronal cells, various histone modifications were mapped across the *Avp* locus and resulted in the identification of high amounts of the repressive histone mark H3K27me3, a hallmark of PcG complexes, concomitant with moderate amounts of active histone marks, at the downstream enhancer (Murgatroyd and Spengler, 2014). The presence of PcG complexes was further corroborated by detection of its catalytic subunit, the histone methyltransferase Suz12, and associated Tet1 and Tet2 proteins, which account for high levels of 5hmC enhancer methylation (Figure 6A).

Conversely, hypothalamic-like differentiation triggered eviction of PcG and Tet proteins, loss of repressive chromatin marks, and a surge in activating histone marks together with Avp expression (Murgatroyd and Spengler, 2014). At the same time, the *de novo* DNA methyltransferase Dnmt3a started to invade and methylate the enhancer region promoting subsequent recruitment of Mecp2. Moreover, in support of a role in instigating DNA methylation, pharmacological depletion of PcG complexes prevented *Avp* enhancer methylation (Figure 6A). Consistent with this view, a recent report showed that the catalytically inactive DNA methyltransferase Dnmt3L interacts with the polycomb PRC2 complex in competition with Dnmt3a and Dnmt3b to maintain low methylation levels at bivalent domains of developmental genes (Neri et al., 2013).

Overall, the *Avp* enhancer matches the criterion of a “bivalent domain” known to facilitate robust transitions in neurodevelopmental gene expression and suggests to its DNA methylation a potential role for fine-tuning *Avp* expression rather than a switch-off.

We previously reported that ELS in mice (period infant-mother separation for 3 h per day from postnatal day (PND) 1–10) causes lasting hyperactivity of the HPA axis marked by heightened corticosterone secretion under resting conditions and hyperresponsiveness to acute stressors applied in later life. These neuroendocrine signs were accompanied by distinct behavioral changes including memory deficits in an inhibitory avoidance task and increased immobility in the forced swim test (Murgatroyd et al., 2009).

AVP plays a leading role for an appropriate adrenocortical response to stress during fetal and perinatal life when the endocrine response to stress is dampened (Murgatroyd and Spengler, 2011), while CRH becomes increasingly important as the organism matures.

In accord with this view, ELS caused a fast and sustained upregulation of *Avp*, but not of *Crh*, in the hypothalamic PVN. Heightened *Avp* expression associated with reduced DNA methylation at the enhancer and was most evident at 6 weeks and 3 months. Expectably, Mecp2 binding was diminished in young adult ELS treated mice when compared to controls but surprisingly also at PND 10 when DNA methylation was still intact. Previous studies have shown that neuronal activity can trigger calcium-dependent phosphorylation of Mecp2, notably at serine 421, causing dissociation from the DNA and derepression of target genes (Bellini et al., 2014). In agreement with the possibility that ELS may trigger derepression of *Avp* via Mecp2 phosphorylation, we detected increased Mecp2-S421 phospho-immunoreactivity in the PVN of newborn ELS-treated mice, but not at 6 weeks, when compared to controls (Figure 6B).

These findings suggest that the rapid increase in Avp expression in response to ELS is mediated by Mecp2’s dissociation from the enhancer due to neuronal-activity driven phosphorylation. Since Mecp2 provides a platform for the recruitment of Dnmts, its absence from the enhancer will expedite DNA demethylation during postnatal methylome reconfiguration (Lister et al., 2013; Figure 6B).

In sum, these experiments show that ELS triggers a rapid switch in the function of the epigenetic reader Mecp2, which translates into the formation of a sustained molecular memory at the *Avp* enhancer contributing to the development of distinct neuroendocrine and behavioral phenotypes. Mechanistically, the molecular epigenetic switch subserving developmental Avp expression is coopted by ELS-dependent programming of the HPA axis in response to ELS (Murgatroyd et al., 2009; Murgatroyd and Spengler, 2011). These findings suggest that molecular epigenetic switches can act in series in a contextual fashion.

Finally, much of the work on molecular epigenetic switches in mammalian systems addressed here refers to methylation changes that occur early on in development, and have long-lived effects well into adulthood (e.g., sexual differentiation, puberty, and ELS). There are, however, many sensitive periods depending upon the question (e.g., embryonic development, adrenarche, adult trauma and stress) (Lupien et al., 2009). It is important to note, that molecular epigenetic switches can also couple to different external or internal signals acting at different time windows on the same substrate. In this respect, expression of Avp within the bed nucleus of the stria terminalis (BNST) of adult rat brains can be dynamically altered by changes in testosterone levels (Auger et al., 2011). Specifically, testosterone withdrawal in response to castration triggered enhanced *Avp* promoter methylation and decreased Avp expression. Interestingly, this increase in *Avp* methylation in the BNST can be prevented by testosterone replacement of castrated animals. Together, these findings suggest that *Avp* methylation status is actively maintained in a region-specific manner in adult brain and tightly tracks changes in testosterone levels.

## Conclusions and Outlook

The concept that epigenetic states are intrinsically plastic has gained increasing apprehension in the past years (Feinberg, 2007; Meaney and Ferguson-Smith, 2010; Hoffmann and Spengler, 2012). Here, we propose that they are also inherently unstable and guide dynamic transitions in cellular or organismal phenotypes.

The identification of bivalent domains provides persuasive evidence for molecular epigenetic switches in gene expression in neurodevelopment and beyond. Once this dynamic equilibrium between activation and repression is tilted, robust “ON or OFF” decisions take place. Examples discussed in this review include neuronal differentiation of ESCs, activation of *Kiss1* at the onset of puberty, and experience-dependent epigenetic programming of *Avp*. Epigenetic switches can operate in series as evidenced by the transition of ESCs into NPCs and beyond and may be also triggered by cues of different quality. For instance, a developmental switch at the *Avp* enhancer serves as matrix for a subsequent environmental switch (i.e., the response to ELS) and both together determine future gene expression and phenotype.

Still, the clear cut effects of molecular epigenetic switches on cellular or animal phenotypes seem to translate into more graded responses in human populations. A plausible explanation for this discrepancy is the high degree of genetic homogeneity in conjunction with well-defined experimental conditions in most cell culture and animals studies. In this respect, genetic heterogeneity and different life-experiences are likely sources for phenotypic variation in response to molecular epigenetic switches in human (Heim and Binder, 2012).

In contrast to ELS, light entrainment represents a less complex but meaningful stimulus for resetting genetically encoded circadian cycles. Plastic changes in DNA methylation showed a remarkable flexibility of this mark and associate with reversible switches in circadian behavior. Future studies are still necessary to elucidate the precise order of epigenetic events in the timing of molecular clocks.

Furthermore, epigenetic “ON or OFF” switches at imprinted genes provide a versatile mechanism for dosage regulation of genes with an important role in balancing stem cell quiescence vs. proliferation. These changes occur in a temporospatial manner through so far incompletely understood mechanism across the genome at several loci (Hoffmann et al., 2014; Daniel et al., 2015) and may be therefore relabeled as “epigenomic switches”.

Lastly, retrotransposition in NPCs provides an intriguing example of an epigenetic-like switch occurring stochastically and/or in response to genomic stress. Activation of retrotransposition results from the expression of developmental TF, which are under the control of bivalent domains. While strengthening the importance of bivalent domains for epigenetic switches, these findings also indicate that their functions may extend to increased flexibility regarding “unanticipated challenges that are not precisely programmed” (McClintock, 1984).

Overall, we suggest that the concept of molecular epigenetic switches illuminates the catalyzing function of epigenetic mechanisms in the mediation between dynamically changing environments and the static genetic blueprint. In the context of Waddington’s epigenetic landscapes of valleys and mountains, molecular epigenetic switches are guiding dynamic changes in gene expression underlying robust changes in cellular and organismal phenotypes. Ultimately, molecular epigenetic switches may also serve to reconfigure Waddington’s epigenetic landscape for better or for worse by lowering the threshold for transitions between distinct developmental trajectories and increasing flexibility regarding unanticipated challenges. Although of potential benefit, such changes are also likely to increase the risk for human disease.

## Author Contributions

All authors contributed to conception and writing of this manuscript.

## Conflict of Interest Statement

The authors declare that the research was conducted in the absence of any commercial or financial relationships that could be construed as a potential conflict of interest.
